# The challenge of community mental health interventions with patients, relatives, and health professionals during the COVID-19 pandemic: a real-world 9-month follow-up study

**DOI:** 10.1038/s41598-022-25297-w

**Published:** 2022-12-05

**Authors:** Carlos Roncero, Armando González-Sánchez, Ángela Pérez-Laureano, Carmen Ortiz-Fune, Sara Díaz-Trejo, Miriam Bersabé-Pérez, María Dolores Braquehais, Javier Pérez-Rodríguez, José Ángel Maderuelo-Fernández, José Antonio Benito-Sánchez

**Affiliations:** 1 Psychiatry Service, University of Salamanca Health Care Complex, Paseo de San Vicente 58-182, 37007 Salamanca, Spain; 2 Psychiatric Unit. School of Medicine, University of Salamanca, Campus Miguel de Unamuno, Calle Alfonso X El Sabio S/N, 37007 Salamanca, Spain; 3 Institute of Biomedicine, University of Salamanca (IBSAL), Hospital Virgen de la Vega, 10ª Planta, Paseo de San Vicente, 58-182, 37007 Salamanca, Spain; 4 Department of Statistics, University of Salamanca, Campus Miguel de Unamuno. C/ Alfonso X El Sabio S/N, 37007 Salamanca, Spain; 5 Galatea Care Programme for Sick Health Professionals, Galatea Clinic, Carrer Palafolls, 17-19, 08017 Barcelona, Spain; 6 Mental Health Research Group, Vall d’Hebron Research Institute, Universitat Autònoma de Barcelona, Passeig Vall d’Hebron, 119-129, 08035 Barcelona, Spain; 7 School of Medicine, Universitat Internacional de Catalunya, Carrer Casanova, 143, 08036 Barcelona, Spain; 8 Gerencia de Atención Primaria de Salamanca, Gerencia Regional de salud de Castilla y León (SACyL), C/Arapiles, 25-33, 37007 Salamanca, Spain; 9 Unidad de Investigación en Atención Primaria de Salamanca (APISAL), Avenida de Portugal, 83, 37005 Salamanca, Spain; 10 Network for Research on Chronicity, Primary Care, and Health Promotion (RICAPPS), S/N, 28029 Madrid, Spain

**Keywords:** Human behaviour, Psychology, Epidemiology

## Abstract

Since the beginning of the COVID-19 pandemic, the need to implement protocols that respond to the mental health demands of the population has been demonstrated. The PASMICOR programme started in March 2020, involving a total of 210 requests for treatment. Out of those subjects, the intervention was performed in 53 patients with COVID-19 without history of past psychiatric illness, 57 relatives and 60 health professionals, all of them within the area of Salamanca (Spain). Interventions were carried out by professionals of the public mental health service mostly by telephone. Depending on clinical severity, patients received basic (level I) or complex psychotherapeutic care combined with psychiatric care (level II). The majority of attended subjects were women (76.5%). Anxious-depressive symptoms were predominant, although sadness was more frequent in patients, insomnia in relatives and anxiety and fear in health professionals. 80% of the sample, particularly most of the health professionals, required a high-intensity intervention (level II). Nearly 50% of the people treated were discharged after an average of 5 interventions. Providing early care to COVID-19 patients, relatives and professionals by using community mental health resources can help to reduce the negative impact of crises, such as the pandemic, on the most affected population groups.

## Introduction

At the end of December 2019, reports of the "Severe Acute Respiratory Syndrome Coronavirus 2" began to appear^1,2^. This infection has had a major impact on health systems and has forced a specific reorganisation of mental health services to deal with the presence of potentially traumatic sustained stressors on patients, their relatives and health professionals^1,3–5^.

The pandemic can produce psychological disruptions in the general population^6,7^, generating anxiety^8^ that varies from 29.29 (moderate degree)^9^ to 8.4% (severe/extreme degree)^10^, with the prevalence of generalised anxiety disorder being 35.1%^11^. Depressive symptoms have also been reported in up to 20.1% of people^11^ is also described that depressive symptoms prevalence in post-COVID-19 ranged from 3 to 12%^12^. It is also registered prevalences from 14.6 to 48.3% of depressive symptoms and a post-traumatic stress disorder prevalences from 7 to 53.8%^13^. The subgroups identified with the highest risk of presenting psychiatric symptoms were women, the elderly, people with chronic illnesses, migrant workers, and students^14^. Ceban et. al^15^ suggest that individuals with preexisting mood disorders are at higher risk of having COVID-19, hospitalization and death because of COVID-19. Other Studies^16^ describes protective factors for mental health include male gender, staying with children, employment, confidence in doctors, and spending less time on health information. Another suggested factor that influences the population are the strict measures enacted by governments to contain the spread of COVID-19^17^. The frequency of clinically-significant depression and/or severe depressive symptoms.

Due to personal risk or even death, the presence of the disease affects patients and their relatives by causing them to experience great fear of the progression of the infection^3^, feelings of loneliness, denial or despair. This can also lead to an increased risk of aggression and suicide in patients^18,19^, as well as complicated grief situations in relatives of deceased patients^1^. There is a risk of developing mental health problems as a consequence of catastrophes or epidemics^20,21^. Among previous epidemics, psychiatric morbidity was suggested in 64% of the survivors^22^, including an increase in suicide rates^23^.

Meanwhile, health professionals are not exempt from the presence of anxiety and depression symptoms^24,25^. A 35.6% prevalence of generalised anxiety disorder is described^11^, with up to 44.7% having anxiety symptoms^26^. Depressive disorders reach up to 50.7%^26^, although the percentage of healthcare professionals suffering from severe symptoms is much lower(0.3%), the degree of anxiety and depression among medical staff is higher than among administrative staff in hospital centres^27^.

Health workers, as a result of the COVID-19 pandemic situation in the last two years, have seen how their working environment has become a source of stress (increased working hours, shortage of protective material…), transforming these workers into a risk group for the development of mental illnesses^28,29^. For this reason, it is advisable to implement preventive measures in physical and mental health for health professionals^30^.

In other countries^31^ an increase in the prevalence of psychological adversity has been observed, although this is not directly related to the incidence of COVID-19 cases in those countries. This can be explained because those countries have experienced other epidemics in the past, which contributes to develop coping mechanisms^32^.

In the same way, the previous presence of physical symptoms^33^, previous medical problems and training in medicine and in self-control and protection measures^34^ are identified as independent predictors of psychological adversity.

There is a greater perception of risk in those professionals who have more direct contact with covid patients (emergency and intensive care doctors)^16,28,35,36^.Research shows that early psychotherapy treatment helps reduce post-traumatic stress disorder, anxiety and depressive symptoms and in preventing chronic psychopathology^37^. Telepsychology has been successfully applied to alleviate the consequences of the COVID-19 pandemic^38–41^. This study aims to examine the mental health care response to COVID-19 infection and its impact on the COVID-19 affected patients, relatives and professionals during the first 9 months^5,42,43^.

## Results

**Figure 1:**
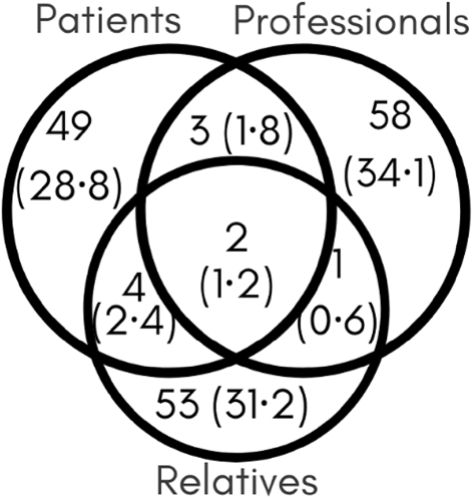
Percentage of each user category based on the survey criteria for classifying patients, relatives of patients and health care staff who treated patients with COVID. Number of users and percentage in parentheses.

There were 210 records over the 9 months, corresponding to 170 people, of whom 53 were patients; 57 relatives and 60 professionals. Nevertheless, combinations of these groups were found (Fig. 1). It included people aged between 5 and 85 years (mean 52.26). The most frequent range was between 51 and 60 years (28.6%). Women predominated (76.5%) accounting for 58.5% of patients, 82.5% of relatives and 86.7% of professionals.

As claimed by the hospital's record history, there were no differences in sex according to the number of cases (U = 35,489.5, p = 0.19), deaths (U = 30,909, p = 0.13) and hospital admissions (U = 30,909, p = 0.84), but there were more male admissions to the ICU (U = 28,787, p < 0.001 d = 0.23).

Among those attended there is a higher proportion of women among professionals and a lower proportion among patients (Chi_2_^2^ = 14.12, p < 0.001,V = 0.28). Women make up the majority of level II users, although this correlation lacks significance (Chi_1_^2^ = 4.32, p = 0.03, Phi = 0.16), and there is no association between age and sex (Chi_7_^2^ = 8.17, p = 0.31). 65.9% of those attended had a partner, 15.9% were widowed, 14.3% were single and 3.0% were separated.

Most of the patients were hospitalised or isolated at home, while most of the relatives or professionals were not hospitalised (Table 1). Treatment was provided to 62 people who had lost a family member or had a relative hospitalised: partner (41.9%), father (16.1%), mother (11.3%), sibling (8.1%), child (4.8%) and others (17.7%).

**Table 1:** User category and employment status, admission, relatives, symptoms, emotions, evolution and intervention.

	COVID Patients	Relatives	Professionals	Total
**Evolution**				
Discharge	27 (50.9)	29 (50.9)	27 (46.6)	83 (49.4)
Monitoring	23 (43.4)	22 (38.6)	25 (43.1)	70 (41.7)
Refused intervention	2 (3.8)	6 (10.5)	4 (6.9)	12 (7.1)
Psychiatry referral	0 (0)	1 (1.8)	3 (5.2)	4 (2.4)
Exitus	0 (0)	0 (0)	1 (1.7)	1 (0.6)
Psychology referral	1 (1.9)	0 (0)	0 (0)	1 (0.6)
Total	53 (100)	57 (100)	58 (100)	168 (100)
**Symptoms**				
Anxiety	33 (63.5)	39 (69.6)	42 (71.2)	114 (68.3)
Insomnia	26 (50)	34 (60.7)	32 (54.2)	92 (55.1)
Crying	23 (44.2)	39 (69.6)	14 (23.7)	76 (45.5)
Emotional blockage	9 (17.3)	21 (37.5)	19 (32.2)	49 (29.3)
Intrusive thoughts	15 (28.8)	20 (35.7)	13 (22)	48 (28.7)
Loss of appetite	11 (21.2)	17 (30.4)	7 (11.9)	35 (21)
Isolation	6 (11.5)	11 (19.6)	15 (25.4)	32 (19.2)
Somatization	7 (13.5)	8 (14.3)	11 (18.6)	26 (15.6)
Conflict	4 (7.7)	9 (16.1)	10 (16.9)	23 (13.8)
Hyperactivity	5 (9.6)	11 (19.6)	6 (10.2)	22 (13.2)
Emotional numbness	3 (5.8)	3 (5.4)	7 (11.9)	13 (7.8)
Thoughts around death	2 (3.8)	2 (3.6)	4 (6.8)	8 (4.8)
Clinophilia	3 (5.8)	5 (8.9)	0 (0)	8 (4.8)
Distrust	3 (5.8)	0 (0)	4 (6.8)	7 (4.2)
Obsessions	6 (11.5)	1 (1.8)	0 (0)	7 (4.2)
Dissociation	3 (5.8)	2 (3.6)	1 (1.7)	6 (3.6)
Confusion	1 (1.9)	1 (1.8)	3 (5.1)	5 (3)
Compulsive acts	1 (1.9)	3 (5.4)	1 (1.7)	5 (3)
Increased appetite	0 (0)	2 (3.6)	1 (1.7)	3 (1.8)
Self-harm	0 (0)	0 (0)	1 (1.7)	1 (0.6)
Suicidal ideation	0 (0)	1 (1.8)	0 (0)	1 (0.6)
Substance abuse	0 (0)	0 (0)	0 (0)	0 (0)
Total	52 (100)	56 (100)	59 (100)	167 (100)
**Relatives**				
No relatives in hospital	52 (94.5)	4 (7.1)	60 (100)	116 (67.8)
Deceased close family members	3 (5.5)	31 (55.4)	0 (0)	34 (19.9)
Hospitalized for more than 7 days	0 (0)	14 (25)	0 (0)	14 (8.2)
Hospitalized for 4–7 days	0 (0)	5 (8.9)	0 (0)	5 (2.9)
Hospitalized for 1–3 days	0 (0)	2 (3.6)	0 (0)	2 (1.2)
Total	55 (100)	56 (100)	60 (100)	171 (100)
**Emotions**				
Sadness	31 (62)	37 (69.8)	27 (45.8)	95 (58.6)
Fear	24 (48)	28 (52.8)	37 (62.7)	89 (54.9)
Impotence	20 (40)	16 (30.2)	17 (28.8)	53 (32.7)
Exhaustion	17 (34)	12 (22.6)	16 (27.1)	45 (27.8)
Lability	15 (30)	5 (9.4)	21 (35.6)	41 (25.3)
Loneliness	8 (16)	12 (22.6)	16 (27.1)	36 (22.2)
Frustration	8 (16)	9 (17)	12 (20.3)	29 (17.9)
Anger	7 (14)	10 (18.9)	10 (16.9)	27 (16.7)
Irritability	6 (12)	6 (11.3)	8 (13.6)	20 (12.3)
Guilt	3 (6)	9 (17)	5 (8.5)	17 (10.5)
Total	50 (100)	53 (100)	59 (100)	162 (100)
**Intervention**				
Abreaction	46 (88.5)	51 (89.5)	41 (68.3)	138 (81.7)
Self-care	34 (65.4)	39 (68.4)	44 (73.3)	117 (69.2)
Time management	19 (36.5)	32 (56.1)	27 (45)	78 (46.2)
Mindfulness	20 (38.5)	18 (31.6)	36 (60)	74 (43.8)
Cognitive therapy	20 (38.5)	21 (36.8)	29 (48.3)	70 (41.4)
Awareness of personal resources	16 (30.8)	33 (57.9)	16 (26.7)	65 (38.5)
Interaction encouragement	13 (25)	25 (43.9)	25 (41.7)	63 (37.3)
Revaluation	10 (19.2)	21 (36.8)	22 (36.7)	53 (31.4)
Bereavement counselling	13 (25)	32 (56.1)	2 (3.3)	47 (27.8)
Scientific information	9 (17.3)	7 (12.3)	11 (18.3)	27 (16)
Prevention of suicidal ideation	1 (1.9)	3 (5.3)	0 (0)	4 (2.4)
Total	52 (100)	57 (100)	60 (100)	169 (100)
**Employment status**				
Sick leave	15 (27.3)	8 (14.3)	30 (50)	53 (31)
Active	4 (7.3)	11 (19.6)	30 (50)	45 (26.3)
Unknown	19 (34.5)	23 (41.1)	0 (0)	42 (24.6)
Retired	17 (30.9)	14 (25)	0 (0)	31 (18.1)
Absenteeism	0 (0)	0 (0)	0 (0)	0 (0)
Total	55 (100)	56 (100)	60 (100)	171 (100)
**Hospitalization**				
Not hospitalized	8 (14.5)	53 (94.6)	58 (96.7)	119 (69.6)
Hospitalized for more than 7 days	19 (34.5)	0 (0)	0 (0)	19 (11.1)
Home isolation	13 (23.6)	2 (3.6)	2 (3.3)	17 (9.9)
Hospitalized for 1–7 days	9 (16.4)	0 (0)	0 (0)	9 (5.3)
Hotel isolation (or similar)	6 (10.9)	1 (1.8)	0 (0)	7 (4.1)
Total	55 (100)	56 (100)	60 (100)	171 (100)

Percentages in parentheses. Data by admission criteria.

The absolute numbers, as well as the percentages, are shown in the table for each type of beneficiary of the program. In this way, the evolution, and emotions of each type of user, the symptoms and interventions, their employment status and whether they have required hospitalization can be observed. Similarly, the table shows whether these users also had affected relatives and what condition they are in.

Of the 60 professionals, half were on leave. Among them were doctors (7.6%), nurses (12.4%), clinical assistants (12.4%), other health professions (carers or orderlies) (9.4%) and other professions such as social work (or others) (58.3%).

Of the total sample, 27.1% were working, 32.9% were on leave, 25.9% were retired and 14.1% whose situation was unknown. Significant differences were found (Chi_2_^2^ = 8.81, p = 0.01, V = 0.29) between patients, relatives and professionals according to their employment status (working or on leave), with the patient group being the only one in which it was more common to be on sick leave (4.5 times more than the other categories). With respect to overall employment status, differences were found in the duration of calls (U = 1648.5, p = 0.01, d = 0.49), as well as the number of calls (U = 1715.5, p = 0.004, d = 0.59), with 1.65 times longer interventions and 1.86 times more calls from those on leave compared to those working.

A total of 927 assistances were provided, most of them by telephone (24 were face-to-face consultations) for a total of 433 h. The face-to-face consultations were carried out with 6 level II health professionals who also treated emotional difficulties and behaviours that were difficult to deal with over the telephone (interpersonal difficulties, difficulties in maintaining attention, high levels of anxiety…). Another of the aims of these consultations was minimising any potential feelings of stigmatisation.

**Figure 2:**
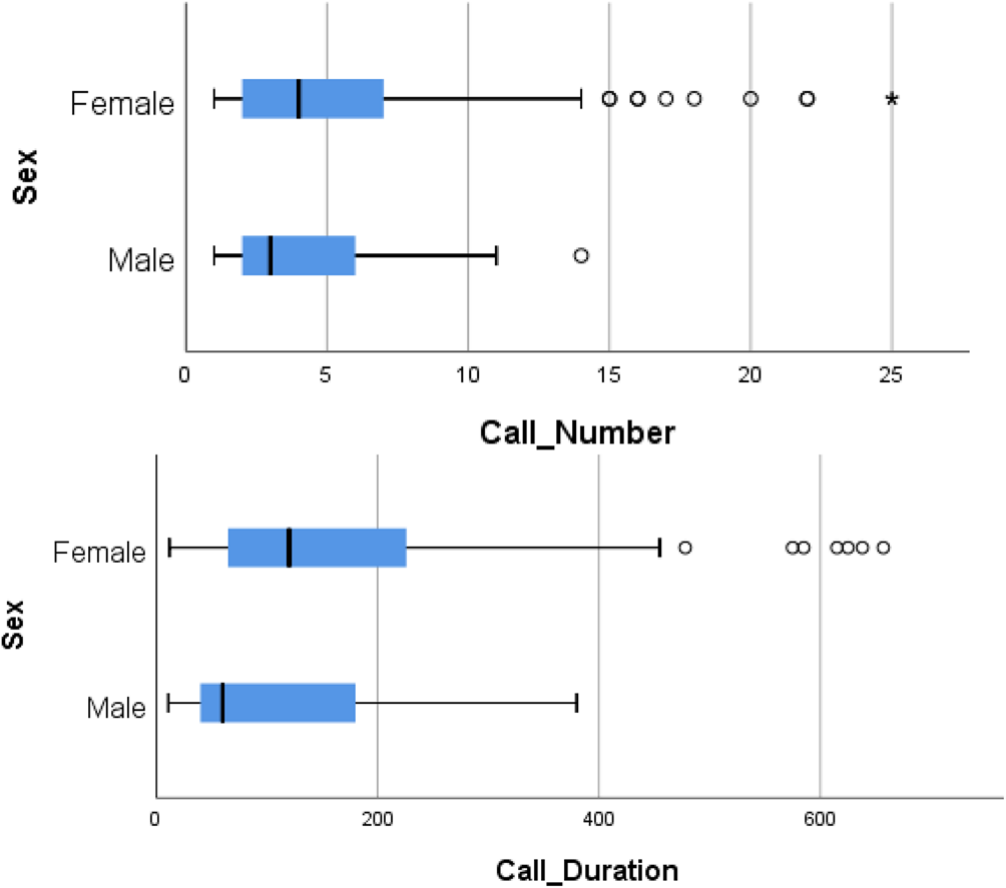
Boxplot of number of calls and duration of the calls, both by sex.

The patient received between 1 and 25 calls with a minimum duration of 11 and a maximum duration of 657 min. No differences were found between the means of the number of calls (Mean = 5.45, SD =  ± 4.83, p = 0.75) and the means of their duration (Mean = 152.71, SD =  ± 137.92; p = 0.60) between patients, relatives and professionals. No differences were found in the number of calls according to sex (U = 2995.5, p = 0.14), but the duration of interventions in women was longer (U = 3352, p = 0.006, d = 0.43) (Fig. 2). Among active, on leave and retired workers, differences were found in the number of interventions (K–W = 9.12, p = 0.01, d = 0.45) but not in the duration (K–W = 5.91, p = 0.05).

The symptomatology shown by the attended users was related to anxiety, insomnia, followed by crying, emotional blockage, intrusive thoughts, loss of appetite and isolation (Table 1). No cases of completed suicide were recorded, but two patients in the relatives' group made two self-harming attempts. Passive ideas of death were reported by 4.8% of people.

In terms of emotions, sadness is the most frequent, followed by fear and helplessness (Table 1). No differences were found among patients, relatives or professionals in terms of emotions (Chi_20_^2^ = 30.51, p = 0.06). There was no difference between the number of discharges, follow-up and refusal in patients, relatives or professionals (Chi_6_^2^ = 2.486, p = 0.87).

Patients were consistently treated, relatives’ treatment was more focused towards resource exposure and bereavement, with fewer treatments involving mindfulness practices; professionals’ treatment was more focused on mindfulness, less on bereavement and not at all on self-harm (Chi_20_^2^ = 51.09, p < 0.001, V = 0.18).

A total percentage of 81.3% were considered level II:77.1% of patients, 72.7% of relatives and 94.2% of professionals, with the latter showing a greater tendency towards level II, while relatives tended to have more level I interventions (Chi_2_^2^ = 8.93, p = 0.01, V = 0.24). Within level II, no further variation was found in terms of interventions (Chi_10_^2^ = 13.2, p = 0.21) or symptoms (Chi_20_^2^ = 18.33, p = 0.56). Although there were more users on sick leave than active users, no differences could be determined in terms of their work activity either (Chi_10_^2^ = 2.73, p = 0.09).

## Discussion

The intervention was carried out on 170 people, mostly with level II intensity. More women were treated in all categories, having longer interventions.

Among the emotions felt, hem ones that stand out are fear, more present in healthcare workers; sadness and helplessness, more present in patients; and fear and sadness in relatives. In terms of the symptoms evaluated, the majority of patients were reported to have symptoms of anxiety and insomnia. The most relevant treatments were techniques related to emotional expression and regulation, and self-care. There is a low incidence of suicide attempts or suicidal ideation. The most frequent interventions are emotional facilitation and self-care, although mindfulness techniques were of particular relevance for professionals. In the case of relatives, both bereavement and resource explanation were significant. Abreaction and self-care were the most common interventions. Interventions had an influence on patients and 46.6% received therapeutic discharge.

As in other studies performed^44–47^, women are more likely to ask for help, to be more vulnerable and to have a worse psychological response to traumatic events, as well as a higher prevalence of disorders and requests for assistance, even though they are less affected by COVID. One of the possible explanations lies in opportunity: people ask for help because they have the opportunity to do so (those who are on sick leave have more time to be treated). However, despite being more affected by COVID, men do not seek more psychological help; in addition, women would be more open socially to express their psychological discomfort than men^44^.

Comparatively, guilt is more prevalent among relatives. Most of the deaths, particularly in the initial weeks of the pandemic, occurred in circumstances of family isolation, with forced separations and the impossibility of performing regular burial ceremonies, resulting in the restriction or impossibility for the patient’s relatives to mourn. Mourning rituals have an emotional value in helping to alleviate feelings of sadness and guilt^48^.

The symptoms detected are consistent with those found in a follow-up study of COVID patients who had been hospitalised, finding a higher prevalence of anxiety and post-traumatic stress symptoms^49^ and with those reported in a recent meta-analysis which highlighted symptoms of anxiety, depression and sleep disturbances as the most common in patients (47%,45% and 34% respectively)^50^. It is also registered in COVID patients impulsivity, insomnia and posttraumatic stress disorder^51^. These results were consistent with the general Spanish population in which symptoms of anxiety, depression and stress were the most prominent in relatives^45^.

The emotional reactions of fear and sadness have already been described^44,52^ along with hyper-preoccupation, sleep disturbances^44^ and references to rational worries^52^. However, as was found in the present study, in many cases health professionals also had ill relatives or had suffered a death in their immediate environment, so they may be influenced through multiple pathways. However, the counterpart of the negative symptoms induces the population to be more per-missive towards vaccination because the high perceived pandemic risk index allows a higher willingness to vaccinate^53,54^.

The absence of suicides, the few attempts detected and the low rates of passive death ideation found differ from the increased suicidality in similar crisis and medical epidemic contexts found in other research^6,18,19,23,55^. These low rates may be due to the success of the programme in improving distress and preventing autolysis. It could also be influenced by the fact that the study has been conducted in the short to medium term and an early clinical assessment has been carried out, with symptoms that may not have fully developed.

The differences found in the higher number of calls in the group who were on sick leave compared to those who were actively working could be explained by the fact that the former have more time to be contacted by the programme and not merely because their cases were more severe, although no other studies were found to support this.

The use of telemedicine understood as remote psychology turned out to be an adequate tool for situations in which the application of face-to-face psychological therapy is complicated, as indicated in other studies^56^. In our study we implemented different types of therapy, but it is studied^57^ that the cognitive behaviour therapy is the most evidence-based treatment, included insomnia^58^, despite its high cost^59^. Fear of contagion of COVID-19, health training, and degree of exposure to the disease appear to be risk factors for suffering from psychological adversity^28,31,34,35^.

**Figure 3:**
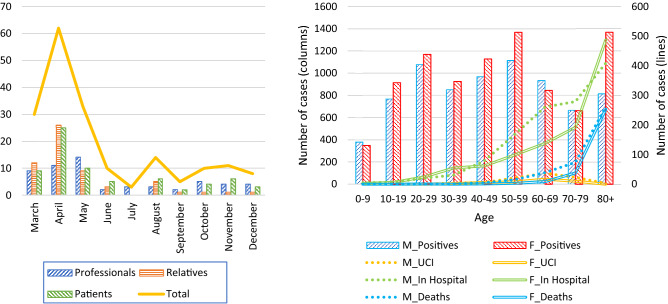
Left: PASMICOR demand evolution by months. Right: Number of hospitalizations, ICU admissions and deaths by sex and age in the province of Salamanca. From 19 March to 30 November. Data obtained from the Ministry of Health^2^. Positives: Number of reported cases confirmed with an active infection diagnostic test (AIDT) as established in the COVID-19 Early Detection, Surveillance and Control Strategy. In Hospital: Number of hospitalised cases. ICU Number of cases admitted to the ICU. Deaths: Number of deaths. (M: male, F: Female).

The need for intervention was mainly found in level II patients. This may be due to the fact that relatives and patients were being referred by other health professionals, and that professionals probably started to demand treatment after an overflow of COVID-19 cases, which is concurrent with the time evolution of the demands (Fig. 3).

The intervention of the CP mainly involved emotional facilitation, along the lines of other studies concerning the need to be able to express negative emotions^43^. This is followed by the prevalence of self-care and cognitive therapy. Among the groups, there were differences due to the greater proportion of this type of intervention in the professionals attended, which perhaps is linked to the differences in the predominant emotions and degree of introspection associated with cultural background. The greater presence of fear and anger may be associated with an increased tendency to use cognitive techniques.

Other intervention programmes were difficult to find, mainly consisting of generic descriptions in published self-help manuals and generic psychological helplines^60^, online interventions targeting at-risk populations^61^, as well as proposals for encouraging social support, remote communication, and emphasising the altruistic value involved in health care as elements of mental health support for health professionals^62^.

In terms of the limitations found in our study, we have to take into consideration that it is a real-world study, with the controls limited to the care target. In addition, remote interventions have posed difficulties when analysing them^62^. This single-centre study was programmed and carried out during COVID times, adapting resources and needs to an unexpected situation, implementing a new method of action (from face-to-face to remote) and using telemedicine.

However, these results should be considered as part of a free and easily accessible clinical care programme. As such, they may help other health services or in the case of a possible recurrence of the pandemic. Knowledge of people's experiences of these situations, both symptomatically and emotionally, along with an understanding of the thematic areas of intervention developed by the clinical psychologists who provided telephone care, can provide guidance on which areas of training should form part of the curricula of health care professionals. Other studies describe generic needs and symptoms but do not provide an analysis focusing on the content of the care provided.

The PASMICOR programme has been a key tool to provide early treatment of the mental disorders without an admission bias. A larger number of women have been assisted in all three groups, with anxiety-depressive symptoms being predominant. Some differences were found, with sadness predominating in patients, insomnia predominating in relatives, and anxiety and fear in professionals. 80% of the sample, particularly most of the health professionals, required a high-intensity intervention. Almost 50% of the people assisted were discharged after an average of 5.0 interventions. This programme prevented the saturation of the community mental health network, and should help to plan and organise care for both the final stages of the pandemic as well as future ones.

## Methods

The Psychiatry Service of the University of Salamanca Health Care Complex (CAUSA) comprises all the mental health resources of the Salamanca area and a relevant part of the drug addiction network. It approximately attends 330,000 inhabitants, to which we must add almost 30,000 university students from other areas^5^.

In order to prevent the long-term consequences of the pandemic, a new Mental Health Care programme was designed and implemented: PASMICOR (Mental Health Care Program for CORronavirus Infection);which started operating in March 2020^42^ to attend health professionals, COVID-19 patients and their relatives with emotional distress and who have requested for help. The programme was disseminated through the CAUSA management and the primary care management. The PASMICOR program is basically exclusive mental health care for population groups especially affected by the pandemic situation. People suffering mental disorders previous to COVID outbreak were attended in the Salamanca mental Health network^63^. This implies that the work of mental health professionals is dedicated exclusively to the subjects who enter this programme.

In order to carry out the project, a team was set up with the regular professionals of the psychiatry service, coming from the inpatient or outpatient units that had been closed or had changed their work procedure. Twelve part-time clinical psychologists, two full-time clinical psychologists and two psychology residents collaborated. There were four psychiatrists for second-level care, which was parallel to the psychological approach. From June 2020 the service was reorganised back into the usual programmes. In addition, three clinical psychologists were recruited specifically for the programme.

The PASMICOR programme did not include patients with previous mental disorders in active treatment nor with hospitalised patients who were critically ill, as they had to be able to communicate with medical staff. It was mainly intended to be a telephonic approach, under the premise of being able to provide a rapid and clinically relevant response that could be assumed by the system and that responded to those situations of greatest need while being safe with regard to the risk of contagion. Relatives’ information was forwarded by e-mail by one of the clinicians responsible for the case. In the case of health professionals, they had the choice to access by sending an email themselves.

**Figure 4:**
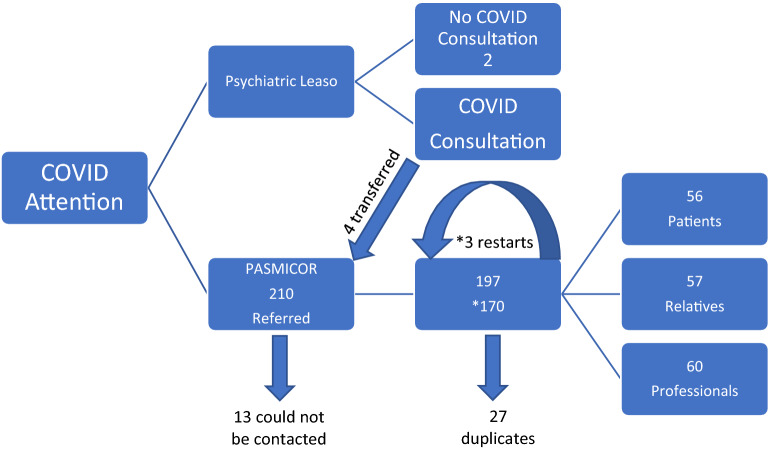
Sample flowchart.

Between 19/3/2020 and 1/12/2020, 210 requests for treatment were received. However, after removing duplicates (27) and the requests of those who we could not contact (13), we narrowed our study to n = 170 (Fig. 4), which indicates the number of people treated on this period. In the event of missing data, the subject was not considered in this variable.

The Clinical Psychologist (CP) initially classified the users by means of a systematic assessment of emotions, symptoms and the psychotherapeutic interventions carried out. The items concerning symptoms, emotions and interventions were established on the basis of the information gathered in the literature^43^, as well as the contributions of the psychologists involved. Clinical judgment was used to measure patient symptoms. The focus of the programme was clearly care-oriented, seeking to provide early psychotherapeutic attention by specialists in clinical psychology. and the need for intervention was categorised into two levels: level I was defined as the least need for care, those demands that involved basic requirements. The need for assistance was categorised into two levels. Firstly, those demands that involved basic requirements with a lower need for assistance were classified as level I cases. Meanwhile, users were categorised into level II when they required more intervention time (more than 20-min calls, excluding the first appointment; more than 3 calls in 1 month per case), greater symptomatic severity a priori (dissociative symptoms, high expressed emotion, etc.), or referral for assistance from other services (group therapy, referral for psychiatric psychopharmacological care, etc.).

This study was conducted in accordance with the guidelines of the Declaration of Helsinki, the appropriate measures have been taken to guarantee the complete confidentiality of the personal data of those participating in this study and in accordance with the "Organic Law 15/1999 of 13 December on the Protection of Personal Data" of the Government of Spain, under the protection of Law 41/2002 and in particular article 8.2 on the use of clinical documentation. Informed consent was obtained from all patients participated in this study. Their questionnaires were anonymized for data processing, separating the identification data of the patient from clinical-care data. The study was presented to the hospital management and with the knowledge of the committee under the code DOC-PSQ-GE-20-01-01 on March 23, 2020. Subsequently, the protocol was updated on March 7, 2020 with the code DOC-PSQ-GE-20-01-02. The data presented in this study are available upon request from the correspondence author. Data are not publicly available due to ethical issues.

A p-value < 0.05 was considered significant. The Shapiro–Wilk test was used as a test for normality and the Mann–Whitney *U* test for non-parametric tests in independent samples. If there were more than two independent samples, the Kruskal–Wallis test was used. Significant tests were performed using the Phi coefficient from Cohen's d statistic or Cramér's *V* as a measure of association in significant tests. For pairs of nominal variables, Chi^2^ was used together with corrected residuals to find out in which cells there is a discrepancy when this value is not within ± 1.96.SPSS was used for the statistical analysis.

### Ethical standards

This project was approved by the Bioethics Committee of the University of Salamanca Health Care Complex. This study was conducted in accordance with the guidelines of the Declaration of Helsinki, the appropriate measures have been taken to guarantee the complete confidentiality of the personal data of those participating in this study and in accordance with the "Organic Law 15/1999 of 13 December on the Protection of Personal Data" of the Government of Spain, under the protection of Law 41/2002 and in particular article 6.2 on the use of clinical documentation.

## Data Availability

Data are available from the correspondence author, upon request. Data are not publicly available due to ethical issues.
